# Presurgical diffusion metrics of the thalamus and thalamic nuclei in postoperative delirium: A prospective two-centre cohort study in older patients

**DOI:** 10.1016/j.nicl.2022.103208

**Published:** 2022-09-20

**Authors:** Marinus Fislage, Stefan Winzeck, Emmanuel Stamatakis, Marta M. Correia, Jacobus Preller, Insa Feinkohl, Claudia D. Spies, Jeroen Hendrikse, Arjen J.C Slooter, Georg Winterer, Tobias Pischon, David K. Menon, Norman Zacharias, on behalf of the BioCog Consortium

**Affiliations:** a Department of Anesthesiology and Intensive Care Medicine, Charité – Universitätsmedizin Berlin, Corporate Member of Freie Universität Berlin, Humboldt-Universität zu Berlin, and Berlin Institute of Health, Berlin, Germany; b BioMedIA Group, Department of Computing, Imperial College London, London, United Kingdom; c University Division of Anaesthesia, Department of Medicine, University of Cambridge, Cambridge, United Kingdom; d MRC Cognition and Brain Sciences Unit, University of Cambridge, Cambridge, United Kingdom; e Addenbrooke’s Cambridge University Hospitals NHS Foundation Trust, United Kingdom; f Witten/Herdecke University, Medical Biometry and Epidemiology Group, Witten, Germany; g Max-Delbrueck-Center for Molecular Medicine in the Helmholtz Association (MDC), Molecular Epidemiology Research Group, Berlin, Germany; h Department of Radiology, University Medical Center Utrecht, The Netherlands; i Department of Intensive Care and UMC Utrecht Brain Center, University Medical Center Utrecht, Utrecht University, Utrecht, The Netherlands; j Department of Neurology, UZ Brussel and Vrije Universiteit Brussel, Brussels, Belgium; k Pharmaimage Biomarker Solutions GmbH, Berlin, Germany; l Max-Delbrueck-Center for Molecular Medicine in the Helmholtz Association (MDC), Biobank Technology Platform, Berlin, Germany; m Berlin Institute of Health at Charité – Universitätsmedizin Berlin, Core Facility Biobank, Berlin, Germany; n Charité – Universitätsmedizin Berlin, Corporate Member of Freie Universität Berlin and Humboldt-Universität zu Berlin, Berlin, Germany

**Keywords:** Neuroscience and Neuroanaesthesia, Postoperative delirium, Thalamic function, Diffusion magnetic resonance imaging, Diffusion kurtosis imaging, POD, Postoperative Delirium, DKI, Diffusion Kurtosis Imaging, FA, Fractional Anisotropy, MD, Mean Diffusivity, FW, Free Water, MK, Mean Kurtosis, Nu-DESC, Nursing Delirium Screening, CAM, Confusion Assessment Method, CAM-ICU, Confusion Assessment Method for the Intensive Care Unit, RASS, Richmond Agitation Sedation Scale, STROBE, STrengthening the Reporting of OBservational studies in Epidemiology, BioCog, Biomarker Development for Postoperative Cognitive Impairment in the Elderly, TR, Repitition Time, TE, Echo Time, MRI, Magnetic Resonance Imaging, MMSE, Mini–Mental State Examination, T1w, T1-weighted, 3T, 3 Tesla, AP, anisotropic power, IDPs, image derived phenotypes, SNR, signal to noise ratio, ZNCC, zero-normalised cross-correlation, QC, quality control, ROI, region of interest

## Abstract

**Background:**

The thalamus seems to be important in the development of postoperative delirium (POD) as previously revealed by volumetric and diffusion magnetic resonance imaging. In this observational cohort study, we aimed to further investigate the impact of the microstructural integrity of the thalamus and thalamic nuclei on the incidence of POD by applying diffusion kurtosis imaging (DKI).

**Methods:**

Older patients without dementia (≥65 years) who were scheduled for major elective surgery received preoperative DKI at two study centres. The DKI metrics fractional anisotropy (FA), mean diffusivity (MD), mean kurtosis (MK) and free water (FW) were calculated for the thalamus and – as secondary outcome – for eight predefined thalamic nuclei and regions. Low FA and MK and, conversely, high MD and FW, indicate aspects of microstructural abnormality. To assess patients’ POD status, the Nursing Delirium Screening Scale (Nu-DESC), Richmond Agitation Sedation Scale (RASS), Confusion Assessment Method (CAM) and Confusion Assessment Method for the Intensive Care Unit score (CAM-ICU) and chart review were applied twice a day after surgery for the duration of seven days or until discharge. For each metric and each nucleus, logistic regression was performed to assess the risk of POD.

**Results:**

This analysis included the diffusion scans of 325 patients, of whom 53 (16.3 %) developed POD. Independently of age, sex and study centre, thalamic MD was statistically significantly associated with POD [OR 1.65 per SD increment (95 %CI 1.17 – 2.34) p = 0.004]. FA (p = 0.84), MK (p = 0.41) and FW (p = 0.06) were not significantly associated with POD in the examined sample. Exploration of thalamic nuclei also indicated that only the MD in certain areas of the thalamus was associated with POD. MD was increased in bilateral hemispheres, pulvinar nuclei, mediodorsal nuclei and the left anterior nucleus.

**Conclusions:**

Microstructural abnormalities of the thalamus and thalamic nuclei, as reflected by increased MD, appear to predispose to POD. These findings affirm the thalamus as a region of interest in POD research.

## Highlights

• The neuronal basis of postoperative delirium is a subject of ongoing research.
• This study used diffusion kurtosis imaging to elucidate the role of the structural integrity of the thalamus prior to surgery.
• Thalamic mean diffusivity was found to be associated with postoperative delirium.
• Thalamic nuclei potentially involved in the etiology of postoperative delirium have been identified.

## 1 Introduction

Postoperative delirium (POD) represents one of the most frequent adverse events after surgery, especially affecting older patients. (Winterer et al., 2018) Episodes of delirium are characterised by, inter alia, transient deterioration in memory, attention, consciousness, and cognition. (European Delirium Association, American Delirium Society, 2014, Wilson et al., 2020) Although the neuronal basis of POD has not been fully understood to date, the thalamus marks a structure in the brain that has been previously linked to POD. (Cavallari et al., 2016) This is not only because the aforementioned neuronal functions partly underlie the regulation of the thalamus and thalamocortical circuits, (Llinás et al., 1998, van der Werf et al., 2003, Halassa and Kastner, 2017) but also because anaesthetics are known to change functional connectivity. (White and Alkire, 2003, Stamatakis et al., 2010) They also reduce the regional blood flow and metabolism in the thalamus. (Alkire et al., 2008, Xie et al., 2011) This may place an additional burden on already vulnerable patients such as older patients with signs of thalamic atrophy. In a previous study, we have shown that patients with smaller thalamic volume are more susceptible for POD, possibly caused by reduced cellular brain reserves. (Fislage et al., 2022) A volumetric surplus may buffer the impact of stressors, e.g. surgery, and thus, act preventive against the development of neurocognitive disorders.

Analogously to macroscopic shrinkage, previous works suggest that preoperative microstructural decline might also increase the risk for POD. For instance, the SAGES (Successful Ageing after Elective Surgery) study group examined the preoperative diffusion characteristics in different brain regions of 136 patients, of whom 29 were subsequently diagnosed with POD.(Cavallari et al., 2016) Lower fractional anisotropy (FA) in the left thalamic hemisphere was associated with the incidence of POD, whereas lower bilateral FA and higher bilateral mean diffusivity (MD) were linked to delirium severity. Furthermore, another study found in 16 patients with POD a lower FA in the left ventral anterior nucleus in comparison to 97 patients, who also underwent surgery but did not present with POD. (Shioiri et al., 2010) A more recent study suggests that higher radial diffusivity and MD were each associated with POD incidence and severity, showing that diffusion analyses are a promising tool in elucidating the neural correlates of POD. (Tanabe et al., 2020) However, all three studies were exploratory in nature and specific thalamic nuclei have not been examined.

We aimed to analyse the association of presurgical microstructural disintegration of the thalamus with the development of POD. Consequently, we examined the diffusion metrics FA, MD, mean kurtosis (MK) and free water (FW) derived from brain MRI images obtained from patients before undergoing elective surgery and examined the association of these parameters to incidence of POD. Low FA and MK and, conversely, high MD and FW indicate microstructural abnormalities. FW is considered to reflect portions of extracellular water, which are possibly related to progressing neuronal atrophy, (Gullett et al., 2020) whereas the metrics FA, MD and MK cannot directly be translated into unique microstructural features of brain tissue. (Scholz et al., 2014) Instead, their synopsis allows an interpretation of characteristics such as axonal integrity, neuronal organisation or abnormally high, undirected diffusivity. We sought to affirm the role of the premorbid thalamus in the development of POD. We further hypothesised that the impaired microstructural integrity of the thalamus predisposes for POD.

As a secondary outcome, we intended to elucidate the role of eight specific thalamic nuclei and compounds in a consecutive exploratory analysis. Because of previously observed association with POD and the extensive corticothalamic connections of these nuclei, as discussed in the precursor study, (Fislage et al., 2022) we expect that in patients with POD presurgical diffusion metrics show alterations in the pulvinar, mediodorsal and ventral anterior nuclei.

## 2 Methods

This paper has been written in accordance with the ‘Strengthening the reporting of observational studies in epidemiology’ (STROBE) checklist.

### 2.1 Study design

This is an analysis based on the EU-funded multi-centre prospective observational cohort study ‘Biomarker Development for Postoperative Cognitive Impairment in the Elderly’ study (BioCog, https://www.biocog.eu). Recruitment took place from October 2014 to September 2019 at two study sites. The study aimed to develop molecular and neuroimaging biomarkers for postoperative cognitive impairment, e.g., POD.(Winterer et al., 2018) The study was approved by the local ethics committees (No. EA2/092/14 in Berlin, Germany and No. 14–469 in Utrecht, Netherlands) and registered on clinicaltrials.gov prior to enrolment (NCT02265263). Patient’s written informed consent was obtained.

**Fig. 1:**
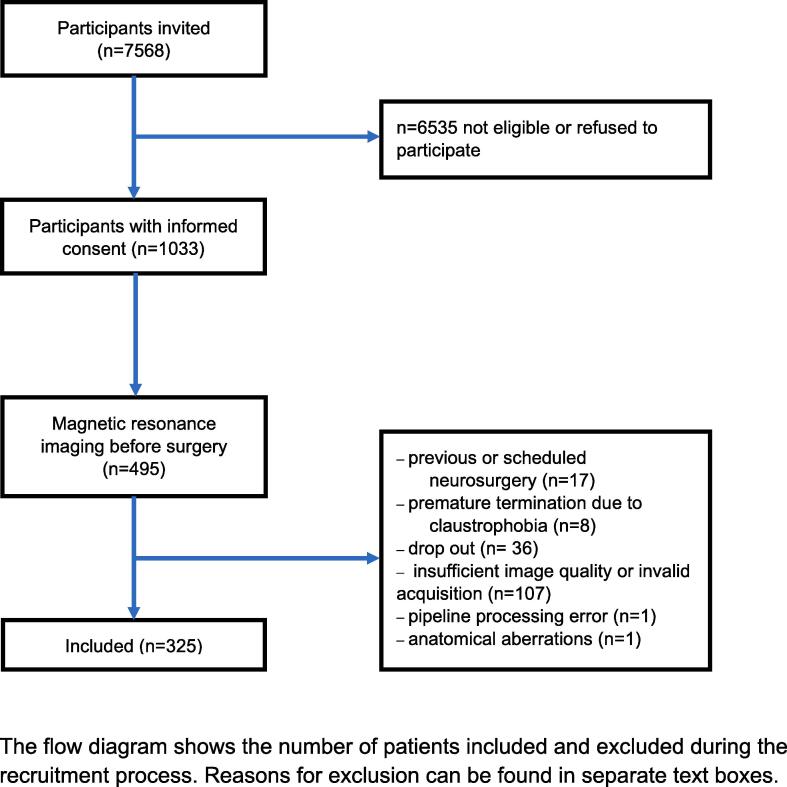
‘Strengthening the Reporting of Observational Studies in Epidemiology’ (STROBE) diagram.

The study focused on older, non-demented patients, who were scheduled for major surgery. Thus, eligibility criteria included age of 65 and older, a Mini–Mental State Examination (MMSE) score of >23 and an anticipated surgery duration of >60 min. Conditions that may distort the neurocognitive test performance such as psychiatric diseases, blindness and deafness led to exclusion. For this DKI study, MRI eligibility was compulsory. Of 1033 patients with informed consent, 495 were scheduled for MRI sessions. The images of 325 were suitable for our DKI analysis with 39 patients from Utrecht and 286 from Berlin (Fig. 1) For further information on inclusion and exclusion criteria please visit clinicaltrials.gov.

### 2.2 Outcome

The POD assessment aligns with the 5th edition of the Statistical Manual of Mental Disorders (DSM-5) by using four validated tests. (Aldecoa et al., 2017) These were: Nursing Delirium Screening Scale (Nu-DESC), Richmond Agitation Sedation Scale (RASS), Confusion Assessment Method (CAM) Confusion Assessment Method for the Intensive Care Unit score (CAM-ICU) and chart review. Study physicians, study nurses or study assistants, who were working under supervision of a study physician, conducted the tests. Training was designed in the format of blended learning and from bench-to-bedside courses.

Delirium status was evaluated once preoperatively and twice a day after surgery. Evaluation sessions were terminated at discharge or after a maximum of seven days.

Criteria for POD were defined at the design stage of the study. Delirium was diagnosed in case of i.) ≥ 2 cumulative points on the Nu-DESC ii.) and/or a positive CAM score iii.) and/or a positive CAM-ICU score and/or evidence of delirium by chart review.

### 2.3 Neuroimaging

Subjects underwent both whole brain T1-weighted (T1w) structural magnetic resonance imaging (MRI) and DKI within two weeks before surgery. Scans were collected at one of two imaging sites (Charité Universitätsmedizin, Berlin, Germany; University Medical Center Utrecht, Utrecht, Netherlands).

Berlin: Subjects were imaged on a 3T Siemens Trio Tim MRI scanner (Siemens Healthcare, Erlangen, Germany). Three dimensional T1w images were acquired with isotropic voxel size of 1.0×1.0×1.0 mm^3^, repetition time (TR) 2500 ms and echo time (TE) 4.77 ms. Diffusion kurtosis imaging included one non-diffusion weighted volume (b = 0 s/mm^2^) and 60 diffusion sensitised directions evenly distributed on two shells (b = 1000, 2500 s/mm^2^). Images were parameterized with TE = 6500 ms, TR = 100 ms and isotropic voxel size of 2.5×2.5×2.5 mm^3^.

Utrecht: MRI scans were collected on a 3T Philips Achieva scanner (Philips Healthcare, Best, The Netherlands). All T1w images were acquired with TR = 7.9 ms, TE = 4.5 ms and isotropic voxel size of 1.0×1.0×1.0 mm^3^. Diffusion kurtosis imaging comprised one non-diffusion weighted volume (b = 0 s/mm^2^) and 60 diffusion sensitised directions evenly distributed on two shells (b = 1000, 3000 s/mm^2^) and acquired with TR = 3294 ms, TE = 68 ms and isotropic voxel size of 2.5×2.5×2.5 mm^3^.

**Fig. 2:**
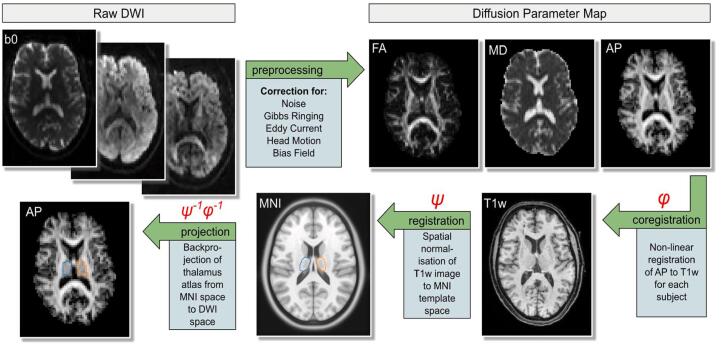
**Schematic Overview of Image Processing.** Note: This schematic overview displays the processing steps for the metrics fractional anisotropy (FA) and mean diffusivity (MD). The processing stream for mean kurtosis (MK) and free water (FW) is not shown. AP = anisotropic powermap. T1w = T1-weighted; 3T, 3. DWI = diffusion weighted imaging. MNI = MNI ICBM152 2009a template.

**Fig. 3:**
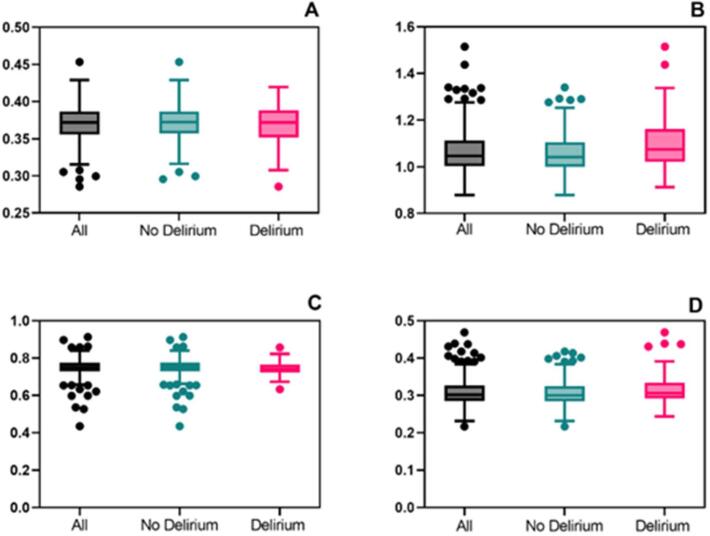
**Boxplots of thalamic diffusionkurtosisimaging metrics across groups.** Note: Boxplots showing the following diffusion metrics within the thalamus across groups: A) Fractional Anisotropy [p = 0.39] B) Mean Diffusivity [p < 0.001] C) Mean Kurtosis [p = 0.53] D) Free Water [p = 0.01]. P-values in square brackets were acquired through an unpaired, two tailed *t*-test comparing patients with and without delirium. The different groups – all patients (ALL), patients without delirium (No Delirium) and with delirium (Delirium) – are listed on the x-axis. Outliers have been defined according to the Tukey method. The y-axis shows the values of the diffusion weighted analysis without any transformation of the numbers. For visualisation purposes, only the values for Mean diffusivity have been scaled by multiplying them with 1000. Colour schemes are supposed to be colourblind safe.

An overview of all imaging protocols is provided in Table 1. The processing pipeline has been visualised in Fig. 2.Fig. 3.Table 2.

**Table 1:** Description of scanning sequence parameters for Berlin and Utrecht setup.

Measure	Scan Parameter Berlin (3T Siemens Magnetom TrioTim)	Scan Parameter Utrecht (3T Philips Achieva)
T1 MPRAGE	192 sagittal slices, 1×1×1 mm^3^ voxel size, TR/TE = 2500/4.77 ms	voxel size = 1×1×1 mm^3^; TR/TE = 7.9/4.5 ms
Resting State fMRI	12 channel head coil; EP2D-BOLD, 8 min duration, 238 slices (first 10 were discarded to reach equilibrium of spin history), descending slice order, voxel size 3x3x3mm^3^, TR/TE = 2000/30 ms, eyes closed	EP2D-BOLD, 8 min duration, 238 slices (first 10 were discarded to reach equilibrium of spin history), descending slice order, voxel size 3x3x3mm^3^, TR/TE = 2000/30 ms, eyes closed
pCASL	voxel size = 3×3×7 mm^3^; TR/TE = 4000/14 ms, label duration = 1650 ms, post labelling delay = 1525 ms	voxel size = 3×3×7 mm^3^; TR/TE = 3919/17 ms, label duration = 1650 ms, post labelling delay = 1525 ms
DWI	50 transversal slices, voxel size = 2.5×2.5×2.5 mm^3^, TR/TE = 6500/100 ms	voxel size = 0.96×1.19×4 mm^3^; TR/TE = 3294/68 ms
High Resolution T1	24 slices, 0.4×0.4×2 mm^3^ voxel size, TR/TE = 8020/48 ms, covering hippocampal volume	Not available

Note: If not stated elsewhere a 32 channel head coil was used.

**Table 2:** Characteristics of patients.

	All N = 325	No Delirium N = 272	Delirium N = 53
Age [years] – mean (SD)	72.3 (4.9)	72.0 (5.0)	73.8 (4.3)
Female Sex	136 (41.8 %)	110 (40.4 %)	26 (49.1 %)
Body Mass Index (BMI) – median (IQR)	26.5 (4.9)	26.5 (4.9)	26.7 (5.1)
	N = 324	N = 271	
Diabetes	71 (22 %)	57 (21.0 %)	14 (26.4 %)
Hypertension	215 (66.2 %)	179 (65.8 %)	36 (67.9 %)
History of Stroke	25 (7.7 %)	18 (6.6 %)	7 (13.2 %)
Malignancy	113 (34.8 %)	89 (32.7 %)	24 (45.3 %)
Preoperative Anaemia	91 (28 %)	74 (27.2 %)	17 (32.1 %)
Mini-Mental State Examination (MMSE) – mean (IQR)	29 (2)	29 (2)	28 (3)
Preoperative Cognitive Impairment	33 (10.2 %)	24 (8.8 %)	9 (17.0 %)
Benzodiazepine Premedication	41 (12.9 %)	33 (12.1 %)	8 (15.1 %)
Duration of anaesthesia [min] – mean (SD)	201.5 (140.2)	177.4 (107.4)	324.4 (208.8)
	N = 323	N = 270	
Type of anaesthesia			
1. General	252 (77.5 %)	218 (80.1 %)	34 (64.2 %)
2. Regional	18 (5.5 %)	16 (5.9 %)	2 (3.8 %)
3. Combined	55 (16.9 %)	38 (14.0 %)	17 (32.1 %)
Type of surgery			
1. Musculoskeletal	100(30.8 %)	85(31.1 %)	15(28.8 %)
2. Gastrointestinal	51(15.7 %)	38(13.9 %)	13(25.0 %)
3. Cardiovascular or thoracic	25(7.7 %)	19(7.0 %)	6(11.5 %)
4. Genitourinary	62(19.1 %)	50(18.3 %)	12(23.1 %)
5. Otorhinolaryngology	25(7.7 %)	24(8.8 %)	1(1.9 %)
6. Oral and maxillofacial	16(4.9 %)	15(5.5 %)	1(1.9 %)
7. Ophthalmology	21(6.5 %)	20(7.3 %)	1(1.9 %)
8. Neurosurgery	7(2.2 %)	7(2.6 %)	0 (0 %)
9. Other	18(5.5 %)	15(5.5 %)	3(5.8 %)
ASA score			
1. ASA I	10 (3.1 %)	9 (3.3 %)	1 (1.9 %)
2. ASA II	214 (65.8 %)	184 (67.6 %)	30 (56.6 %)
3. ASA III	101 (31.1 %)	79 (29.0 %)	22 (41.5 %)
Length of Stay [days] – median (IQR)	6 (6)	4.5 (5)	12 (17)
Inhouse Mortality	3 (0.9 %)	2 (0.7 %)	1 (1.9 %)

*Note:* The table shows characteristics of all participants, the non-delirious and the delirious group. For categorical variables percentages are given instead of mean and standard deviation (SD) in parentheses. Percentages refer to the proportion of the corresponding group (all; No Delirium; Delirium). The N of patients with available data was added to items with cases of missing data. (ASA score ≙ American Society of Anesthesiologists’ Physical Status Classification; IQR ≙ Interquartile Range ≙ 25th to 75th percentile).

### 2.4 Preprocessing

Magnetic resonance images were processed with an in-house designed pipeline. All T1w images were corrected for field inhomogeneities (Tustison et al., 2010) and subsequently non-linearly spatially normalised (Avants et al., 2009) to the non-linear symmetric MNI ICBM152 2009a template. (Louis, 2015) Diffusion weighted images were first denoised via MPPCA, (Veraart et al., 2016) and then corrected for Gibbs ringing artefacts, (Kellner et al., 2016, Henriques et al., 2017) eddy current distortions as well as head motion (Andersson and Sotiropoulos, 2016) and lastly field inhomogeneities. (Jeurissen et al., 2014) Diffusion kurtosis tensors (Jensen et al., 2005) were fitted with dipy (Garyfallidis et al., 2014) to compute FA, MD and MK maps. Furthermore, anisotropic power (AP) maps (Dell’Acqua, et al., 2014) as well as FW maps were calculated.(Louis, 2015) Each AP was non-linearly coregistered to a subject’s corresponding T1w image.(Avants et al., 2009)

### 2.5 Extraction of image derived phenotypes

Both transformations for coregistration (AP to T1w) and spatial normalisation (T1w to MNI) were inversely applied to project the thalamus atlas defined by Najdenovska et al. from MNI space to subject-specific DKI space. (Najdenovska et al., 2018) Thereby, voxel intensities were linearly interpolated which provided certainty estimates for the voxels belonging to the particular regions. Based on these certainty values, weighted averages for different diffusion parameters (i.e. FA, MD, MK and FW) were calculated for each of the 15 regions (entire thalamus and 14 thalamic nuclei). The entire thalamus region was composed of all 14 regions and embraced thalamic structures from both brain hemispheres. This allowed to account for partial volume effects as boundaries of regions, with lower certainties, had a lower impact on the regional average diffusion metrics. In addition, outlier voxels with FA > 1 or λ1 < 0 were excluded from computing the regional mean diffusion metrics. With 15 regions of interest (ROIs) and four diffusion metric maps, we yielded 60 image derived phenotypes (IDPs).

### 2.6 Quality Assessment

The automated preprocessing pipeline also provided quantitative quality control (QC) metrics. These included the number of outliers within thalamus (outliers were defined as voxels with values of FA > 1 or λ1 < 0), signal to noise ratio (SNR) within thalamus, the average head motion between consecutive MR volume acquisitions as well as the similarity between aligned images. The quality of spatial normalisation was assessed by computing the zero-normalised cross-correlation (ZNCC) between warped T1w images and the MNI T1w atlas within the template brain mask. Likewise, similarity between coregistered APs and T1w images within T1w brain mask were estimated for each subject. The ZNCC was computed as.

$$ZNCC\left(x,y\right)=\frac{1}{N}\sum_{i=1}^{N}\frac{\left(xi-\mu \left(x\right)\right)\left(yi-\mu \left(y\right)\right)}{\sigma \left(x\right)\sigma \left(y\right)}$$

With images × and y to be compared and the number of voxels N within the brain mask, μ and σ represent the images mean values and standard deviations, respectively. The SNR was computed by dividing the mean signal by the standard deviation (SD) of the noise. Both metrics were derived from the first b0 image within the thalamus after denoising (noise map is the difference between raw and denoised image).

The five QC metrics were compared across patients with and without POD (no-POD). One patient showed a much lower ZNNC score for its spatially normalised T1w image (ZNNC = 0.67) than all other patients (ZNNC > 0.72). This suboptimal spatial normalisation, caused by enlarged ventricles, may negatively affect the thalamic segmentation. Hence, this patient was excluded from any further analysis. For the remaining patients the ZNNC between T1w image and MNI T1w atlas image was statistically equal between both groups (no-POD: median[IQR] = 0.813[0.029] POD: median[IQR] = 0.807[0.026]; Mann-Whitney *U* test: p = 0.0534). Likewise, the ZNCC scores between AP map and T1w images were similar for the two patient groups (no-POD: median[IQR] = 0.855[0.026] POD: median[IQR] = 0.860[0.030]; Mann-Whitney *U* test: p = 0.0609). The majority of patient scans did not show any outlier (no-POD: 73.9 %, POD: 79.2 %). Less than eight outlier voxels were found within the thalamus for both patient groups (no-POD: 7, POD: 4), which equates to<0.7 % of the entire volume thalamus (outlier voxels were excluded, see above). No statistically significant difference was found in SNR within the thalamus for patients without and with POD (no-POD median[IQR] = 263.5 [77.4]., POD: median[IQR] = 269.4 [68.2]; Mann-Whitney *U* test: p = 0.4). Furthermore, the average head motion for both patient groups was statistically comparable (no-POD median[IQR] = 0.17[0.11], POD: median[IQR] = 0.15[0.12]; Mann-Whitney *U* test: p = 0.39). (see Supplements).

Lastly, mosaic axial images of all FA maps with outlined thalamus segmentations were blindly in regards of delirium status, visually inspected by two independent raters. Neither strongly degraded image quality nor severe errors of thalamus segmentations could be observed, thus all scans were retained. After the QC, the analysis included 325 patients (Berlin: n = 286, Utrecht: n = 39), and 15 healthy subjects scanned at both imaging sites (travelling heads). Patients with insufficient data quality due to acquisition problems, artifacts or QC were excluded.

### 2.7 Statistical analysis

Since DKI metrics do not have specific SI units and we also aimed to compare effect sizes across different metrics, we decided on z-standardising the DKI results before analysis. Consequently, effect sizes are presented as odds ratio (OR) per increment of one standard deviation. Missing data, e.g., due to insufficient image quality, was assumed to be missing at random. Hence, we chose to perform complete case analyses.

For the primary outcome, each DKI metric was considered in a separate multivariable logistic regression. In total, four hypotheses were tested. We accounted for multiple testing by applying a strict Bonferroni correction. The initial significance level of p < 0.05 was set to p < 0.0125. The consecutive analysis of nuclei was exploratory in nature. Hence, a significance level was not predefined, and p-values were omitted. However, structures were considered to be regions of interest based on confidence intervals (CI) that did not include 1. Although this is derived from conventional assumptions of statistical significance, we refrained from statements about significance in the secondary analysis. Not least because there were no corrections for multiple testing applied.

Only adjusted results were considered for interpretation. We also listed the unadjusted results to make changes visible, which were caused by adjusting. Covariates were selected prior to analysis. Covariates used for adjustment were age, sex, and study site. Age was added to the logistic regression due to its potential confounding effect since it affects thalamic DKI metrics and the risk for POD alike.(Winterer et al., 2018) (Fama and Sullivan, 2015) The variable sex served as a potential confounder. By including the study site as binary variable, we accounted for potential deviations from the different scanners and imaging protocols conducted. To explore the dependence structure of the covariates, for the variable age a Mann-Whitney-U and for the variable sex a Chi^2^ test was administered to compare the specifics between the group that developed delirium and the group that did not develop delirium. To ensure that patients whose DKI data could not be assessed, profoundly differ from those patients we eventually included, we also compared the variables age (*t*-test), sex, MMSE score and POD status (both Chi^2^).

Presuming that anaesthetic agents may represent a stressor for the thalamus, we additionally compared the duration of anaesthesia between groups. After differences in terms of duration of anaesthesia between groups became evident, we decided to perform a post-hoc sensitivity analysis. We aimed for determining a potential confounding role of covariates related to the surgical procedure. To this end, we expanded our predefined models with variables that were related to the surgical and anaesthesiologic procedures. Thus, we included the variables duration of anaesthesia, type of anaesthesia (general, regional or combined) and type of surgery to the whole thalamus analysis.

Statistical analyses were carried out on GraphPad Prism (version 9.3.1.). Colour blindness accessibility of graphs and figures was ensured by using colourblind safe colour schemes. For visualisation purposes, we used an unpaired, two-tailed *t*-test to compare whole thalamus metrics between patients with and without delirium.

## 3 Results

The final analysis sample with complete data on pre-operative brain imaging included 325 patients; 39 were from Utrecht and 286 were from Berlin. (For Comparability check please see above). There were 109 patients, whose data was excluded due to insufficient image quality. Those patients whose DKI data we could analyse did not differ in respect to age, sex, MMSE score and delirium status from those, who received a presurgical MRI but were not eligible for this analysis or whose DKI data was invalid. (Supplemental Table 1) After surgery, a total of 53 patients developed POD (16.3 %) during their stay in hospital. The patient group with POD was significantly older in median than the patient group without POD (no-POD: median = 71.0; POD: median = 74.0; difference −3 [CI 95 % −3 – (-1)] Mann-Whitney-U: p = 0.003). Out of a total of 136 female patients participating, 26 (19.12 %) developed POD. Of 189 males in total, 27 (14.29 %) were affected. Although female patients were proportionally overrepresented in the delirium group, the association of sex with POD was not statistically significant (Chi^2^ p = 0.25). The duration of anaesthesia was significantly longer in patients that later had POD (POD: mean 324.4 min versus No-POD: 177.4 min) showing a mean difference of 147.0 min (two-tailed unpaired *t*-test; p < 0.001)(Table 1).

The primary outcome focused on the association between four different DKI metrics measured in the thalamus with POD. Accounting for age, sex and study site as confounding factors, MD was significantly associated with the incidence of POD [OR 1.65 per SD increment (95 %CI 1.17 – 2.34) p = 0.004]. The DKI metrics FA (p = 0.84) and MK (p = 0.41) were not significantly associated with POD. FW showed a significant association in unadjusted analysis with a p-value below the corrected alpha-level [OR 1.42 per SD increment (95 %CI 1.08–1.87) p = 0.01]. However, after adding the adjusting covariates to the regression model, no statistically significant difference was observed [FW (p = 0.06)]. (Table 3)(Fig. 2).

**Table 3:** Thalamic Diffusion Tensor Imaging Metrics and their Association with Postoperative Delirium.

	OR (95 % CI)	p-value	OR adjusted (95 % CI)	p-value
Fractional Anisotropy	0.88 (0.66–1.18)	0.39	1.04 (0.72–1.50)	0.84
Mean Diffusivity	**1.60 (1.22**–**2.12)**	**<0.001**	**1.65 (1.17 – 2.34)**	**0.004**
Mean Kurtosis	0.91 (0.69–1.23)	0.53	0.88 (0.65–1.22)	0.41
Free Water	**1.42 (1.08**–**1.87)**	**0.01**	1.43 (0.98–2.11)	0.06

Note: Table showing the results of multivariable logistic regressions, each of them with a different diffusion tensor imaging metric as predictor variable and their odds ratio (OR) for postoperative delirium. Effect sizes are given as odds ratio (OR) per standard deviation increment. 95 % confidence intervals (95 % CI) are shown in brackets. Adjusted multivariable logistic regression (OR adjusted) was run together with the adjustment variables age, sex and study site. After a Bonferroni correction the significance level was set to p < 0.0125. Significant results are in bold numbers.

We explored DKI metrics in thalamic nuclei or thalamic regions in secondary analyses. In these analyses, for MD, alterations in the following nuclei and compartments were associated with POD: thalamic bilateral hemispheres [OR per SD increment; left 1.59 (95 % 1.14–2.21); right: 1.60 (95 % CI 1.13–2.26)], pulvinar nuclei [OR per SD increment; left 1.38 (95 % 1.01–1.88); right: 1.41 (95 % CI 1.03–1.93)], mediodorsal nuclei [OR per SD increment; left 1.43 (95 % 1.05–1.94); right: 1.48 (95 % CI 1.05–2.09)], and the left anterior nucleus [OR per SD increment; left 1.40 (95 % 1.02–1.94)]. While the left and right ventral-latero-dorsal regions were observed to have a considerable association with POD, the potential effect did not remain after adjusting for age, sex and study site. Central lateral, lateral posterior and medial pulvinar nuclei were combined to form a single thalamic compartment. Together with the ventral-anterior and the ventral-latero-ventral compartment, they did not present with inclining CI (Table 4).

**Table 4:** Mean diffusivity in thalamic nuclei and their association with postoperative delirium.

Mean diffusivity	Left				Right			
	OR	95 % CI	adj	95 % CI	OR	95 % CI	adj	95 %CI
hemisphere	1.59	1.20–2.10	1.59	1.14–2.21	1.57	1.19–2.07	1.60	1.13–2.26
Pulvinar	1.44	1.09–1.91	1.38	1.01–1.88	1.49	1.11–1.98	1.41	1.03–1.93
anterior	1.46	1.10–1.93	1.40	1.02–1.94	1.29	0.97–1.71	1.15	0.81–1.64
Medio-dorsal	1.46	1.12–1.90	1.43	1.05–1.94	1.47	1.11–1.95	1.48	1.05–2.09
Ventral-latero-dorsal	1.33	1.01–1.74	1.23	0.92–1.63	1.34	1.03–1.75	1.21	0.90–1.64
Central-Lateral/Lateral-Posterior/Medial-Pulvinar	1.28	0.99–1.65	1.22	0.93–1.60	1.27	0.97–1.65	1.20	0.89–1.64
ventral-anterior	1.30	0.997–1.69	1.17	0.88–1.56	1.29	0.99–1.68	1.16	0.85–1.58
Ventral-latero-ventral	1.24	0.95–1.63	1.17	0.88–1.55	1.19	0.91–1.57	1.10	0.82–1,47

Note: Table showing the results of multivariable logistic regressions, each of them with the mean diffusivity of different thalamic nuclei or thalamic regions obtained by diffusion tensor imaging. Effect sizes are given as odds ratio (OR) per standard deviation increment and separately for each hemisphere (Left; Right). 95% confidence intervals (95% CI) are shown in columns. Adjusted multivariable logistic regression (adj) was run together with the adjustment variables age, sex and study site.

Unadjusted CIs of FW indicate a potential effect of a few regions on POD. These were bilateral hemispheres, right pulvinar and bilateral mediodorsal nuclei. However, this was not observed in the adjusted logistic regression analyses. (Supplemental Table 4) For FA and MK none of the nuclei or regions showed a considerable potential association. (Supplemental Tables 2 and 3).

We could use the data of 323 patients for the sensitivity analysis. For two patients the data on the duration of anaesthesia was missing. After performing the sensitivity analysis our results remained largely unaltered. (Supplemental Table 5) Independent of age, sex, study site, duration of anesthesia, type of anesthesia and type of surgery, the OR for MD exceeded the primary results [OR per SD increment 1.84 (95 % CI 1.22–2.76) p = 0.003]. Again, the results for FW were above the corrected alpha-level [OR per SD-increment 1.72 (95 % CI 1.09–2.73) p = 0.02]. FA (p = 0.55) and MK (p = 0.07) remained not statistically significantly associated with POD.

## 4 Discussion

In this prospective observational cohort study of older surgical patients, a higher presurgical MD in the thalamus was associated with a higher incidence of POD. The mean diffusivity describes different aspects of tissue properties such as undirected diffusion in a certain brain compartment. Generally, the higher the MD, the more likely some sort of underlying neuronal impairment must be presumed. In contrast to that, other indicators of microstructural integrity such as thalamic FA, MK and FW were not significantly associated with POD in our study sample. The observed association between thalamus FW with POD was only present in the unadjusted regression. After correcting for age, sex and study site, the association was no longer statistically significant. Since the proportion of FW increases with the age of patients (Gullett et al., 2020), subtle differences between the groups might have been mitigated after correcting for age. Our results remained stable after applying a sensitivity analysis, where we added potential surgery-related confounders such as type of surgery, type of anaesthesia and duration of anaesthesia to the existing regression models.

This pattern also coincides with the findings of our secondary outcome: higher MD in certain nuclei was associated with higher odds of POD. Taking the calculated confidence intervals into account, these nuclei and compartments were bilateral hemispheres, pulvinar nuclei, mediodorsal nuclei, and the left anterior nucleus. As with the DTI metrics for the full thalamus, we did not observe a relevant association of the nuclei’ FA, MK and FW with POD.

Our findings align in parts with previously conducted studies. We were able to transfer the volumetric findings from the precursor study to the DKI setting (Fislage et al., 2022). In a DKI analysis of the SAGES study group, MD in the thalamus was associated with risk and to an even greater extent with severity of POD (Cavallari et al., 2016). In the same study, an impact of thalamic FA on POD was reported. The same applies for Shiori et al. After correcting for age, the FA in the left ventral anterior nucleus was linked to POD (Shioiri et al., 2010). In contrast to those FA-related findings, FA was not significantly associated with POD in our cohort. This might be due to different sample specifics across the different studies such as disparities in age or the time period for the scans before surgery for instance. The SAGES imaging cohort was four years older in mean (76 vs 72 years) and image acquisition took place within a longer time period before surgery (four weeks vs two weeks). Furthermore, the cited studies present a variety of limitations. Shiori et al., solely examined FA. The registration of the diffusion weighted imaging appears to be deficient, and a very large smoothing kernel resulted from the very low imaging resolution (slice thickness = 5 mm). For the SAGES study group, Cavallari et al. pointed out that an inherent bias may underlie subsamples that were assigned for the imaging branch. These patients tend to be cognitively more apt and more resilient in comparison to other subgroups within the study, resulting in smaller event rates. Consequently, neuroimaging subsamples such as the SAGES imaging group are usually underpowered. (Cavallari et al., 2022) In contrast, our study represents the largest of its kind and a state-of-the-art DKI acquisition, protocol and processing was administered. Thus, it might be expected that the previously described associations of thalamic FA and POD incidence are not reproducible and the microstructural disintegration might be limited to diffusivity alterations. The contradicting result may also be caused by the nature of the FA metric. FA can be linked to the fibre integrity of cerebral white matter regions; however, it might not show the same sensitivity in grey matter regions such as the thalamus. (Hansen et al., 2013) However, even in whole-brain structural connectivity analyses Tanabe et al. did not detect an association between FA and POD severity either (Tanabe et al., 2020). This may support our interpretation in contradicting FA findings.

Several clinical studies, including this one, implicate a role of the thalamus in POD. Little is known about the pathophysiological and cellular basis of this notion. In the context of anaesthesia the thalamus was perceived as “consciousness switch”, mainly due to observations made during functional imaging studies (Alkire et al., 2008). However, this attribution appears to be reductionist and it ignores the many mechanisms that lead to the complex phenomenon of anaesthesia. (MacDonald et al., 2015) Thalamocortical disconnection might represent one of them (White and Alkire, 2003). The thalamus is a central relay within the diencephalon and, therefore, presents with extensive connections to cortical areas. Hence, it may also play an important role in brain network disconnection, which has been frequently discussed as potential pathomechanism of POD. (Sanders, 2011, van Montfort et al., 2019) Furthermore, the examined nuclei compartments help to indirectly identify cortical structures that might be involved in the incidence of POD. As a result of our exploration of nuclei compounds, the bilateral pulvinar and mediodorsal nuclei appear to be regions of interest. Their neurons project into the prefrontal cortex. For instance, by pharmacogenetically suppressing the mediodorsal thalamus in mice, thalamofrontal disconnection and impaired performance in prefrontal cognitive tasks was observed. (Parnaudeau et al., 2013) Pulvinar and mediodorsal nuclei may be involved in the regulation of cognitive domains such as attention, decision making and working memory. (Rengel et al., 2018) Those are cognitive functions, which are often impaired during episodes of POD. (Subramaniyan and Terrando, 2019)

Another possible pathomechanism is linked to neuroinflammation as a possible cause of POD. (Mahanna-Gabrielli et al., 2019) The premorbid thalamus, marked by increased diffusivity and thus structural impairment, might be particularly vulnerable to diffuse *peri*- or postoperative inflammation in the central nervous system. Here, the duration of anaesthesia may suggest the extent or complexity of the surgery and, therefore, could display the difference in dose of neuro-inflammation associated insult. Apart from neuroinflammation, other potential pathomechanisms have been discussed. In this context, the discussion of abnormal neurotransmission, cytokine release due to a vegetative stress response and demasking of pre-existing dementia, should be mentioned (Mahanna-Gabrielli et al., 2019). Either way, it seems plausible that one or a combination of some of the aforementioned pathomechanisms may explicitly affect the thalamus. Both basic research and clinical trials, which take the possible effects of surgery and anaesthesia on the thalamus into account, are needed to support the understanding of the neuronal aetiology of POD. Furthermore, we would like to point out that the thalamus represents a structure among others that are potentially involved in the development of POD. Hence, to investigate the magnitude of different effects and their relation to each other, future studies should also consider a whole brain analysis.

There is a need to clarify in which way the nuclei we identified, differ from the rest of the thalamus. A more sophisticated understanding of which nuclei are involved in the aetiology of POD might not only increase our pathophysiological understanding but could also allow a more precise prediction of POD in the future. Furthermore, in case there are some unique properties of the identified structures such as different receptor types, this may help to adapt the selection of anaesthetic agents and could help to conceive treatment options.

### 4.1 Limitation

This study comes with several limitations. For instance, it needs to be expected that the neuroimaging selection bias, which was described for the SAGES study may also apply for the BioCog framework since participation in the neuroimaging analysis was voluntary and patients were assigned according to their personal preferences and availability (Cavallari et al., 2022). Perhaps, there are more patients within the imaging cohort that tend to be more resilient against the stressors linked to surgical procedures. Including more of those patients may cause a mitigation of the initial effect that was observed in the POD group.

The sample size was not explicitly specified for this secondary analysis and had to be adapted due to lower incidence rates than initially presumed. Furthermore, sample sizes across study centres were fairly asymmetric in a way that the Berlin cohort strongly outweighed the Utrecht cohort by 247 (286 vs 39) patients. This was aggravated since 107 patients from Utrecht could not be included due to an image acquisition problem. Thus, we were unable to analyse data from all patients who underwent MRI, which may have introduced a selection bias. However, we found that patients who were included in our analysis did not differ on important sociodemographic and clinical parameters from those who were not, so that we deem an influence of selection bias minimal.

The anaesthesiological management has not been standardised. Yet, depth of anaesthesia was monitored by intraoperative electroencephalogram. However, we could not account for potential effects that arose from the intraoperative handling. Interrater reliability for the POD testing was not determined. Thus, potential deviations in delirium ratings are possible.

We did not study whole brain effects. Thus, we cannot draw conclusions on the interaction between other regions of the brain and the thalamus in the development of POD. The secondary analysis of thalamic nuclei was exploratory. The results of these secondary analyses need to be interpreted cautiously given that we performed multiple tests and did not adjust the confidence intervals for multiple testing. Nonetheless, it may help to identify thalamic nuclei of interest for future studies, where a different sample and dataset are needed to test our hypothesis.

## 5 Conclusion

Preoperative microstructural abnormalities revealed by DKI predispose to an increased risk of POD in older patients. Specifically, the thalamic MD was associated with POD – independently of age, sex and study site. In patients with POD, preoperative MD alterations could also be observed in bilateral hemispheres, pulvinar nuclei, mediodorsal nuclei, and the left anterior nucleus. However, FA, MK and FW in thalamus were not associated with POD in this cohort. Notwithstanding, considering the thalamus as a structure that mainly consists of grey matter, based on the increased MD values, microstructural abnormalities can be presumed. These findings may help to delineate the neuronal aetiology of POD.

## CRediT authorship contribution statement

**Marinus Fislage:** Conceptualization, Data curation, Formal analysis, Investigation, Methodology, Validation, Visualization, Writing – original draft, Writing – review & editing. **Stefan Winzeck:** Conceptualization, Data curation, Formal analysis, Investigation, Methodology, Software, Validation, Visualization, Writing – original draft, Writing – review & editing. **Emmanuel Stamatakis:** Conceptualization, Investigation, Writing – review & editing. **Marta M. Correia:** Conceptualization, Methodology, Supervision, Writing – review & editing. **Jacobus Preller:** Conceptualization, Writing – review & editing. **Insa Feinkohl:** Supervision, Writing – review & editing. **Claudia D. Spies:** Funding acquisition, Project administration, Resources, Supervision, Writing – review & editing. **Jeroen Hendrikse:** Supervision, Writing – review & editing. **Arjen J.C Slooter:** Project administration, Resources, Supervision, Writing – review & editing. **Georg Winterer:** Funding acquisition, Project administration, Resources, Supervision, Writing – review & editing. **Tobias Pischon:** Funding acquisition, Investigation, Supervision, Writing – review & editing. **David K. Menon:** Conceptualization, Resources, Supervision, Writing – review & editing. **Norman Zacharias:** Project administration, Supervision, Writing – review & editing.

## Declaration of Competing Interest

The authors declare that they have no known competing financial interests or personal relationships that could have appeared to influence the work reported in this paper.

## Data Availability

We support the idea of open data. The preoperative data used for the current publication are part of the publicly available BioCog dataset via EBRAINS Data and Knowledge services (www.ebrains.eu)
