# Supplementary material for: Presurgical diffusion metrics of the thalamus and thalamic nuclei in postoperative delirium: A prospective two-centre cohort study in older patients
Source: Neuroimage Clin. 2022 Sep 20;36:103208. doi: 10.1016/j.nicl.2022.103208 (PMC9668602; doi:10.1016/j.nicl.2022.103208)
Supplement: Supplementary data 1 [file mmc1.docx]

**Supplemental Figure 1 – Quality assessment across patients with and without postoperative delirium (POD)**

**
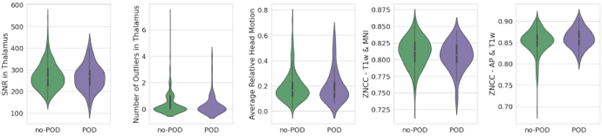
**

Note: Violin Plots display the number of outliers within thalamus (outliers were defined as voxels with values of FA>1 or λ1<0), signal to noise ratio (SNR) within thalamus, the average head motion between consecutive MR volume acquisitions and the similarity between aligned images between POD and No-POD patients. The quality of spatial normalisation was assessed by computing the zero-normalised cross-correlation (ZNCC) between warped T1w images and the MNI T1w atlas within the template brain mask.

**Supplemental Table 1 - Characteristics of included patients (Included) and of patients with invalid or missing DKI data (Excluded)**

| Variable | **Included**  **=325** | **Excluded**  **=159** |  |
| --- | --- | --- | --- |
| Age – mean (SD) | 72.31 (4.94) | 71.79 (4.82) | **P = 0.27^1^** |
| Sex – female/male | 136/189 | 56/103 | **P = 0.16^2^** |
| Mini-Mental-State-Examination (MMSE) – mean (SD) | 28.55 (1.38) | 28.57 (1.40) | **P = 0.86^1^** |
| Postoperative Delirium – number (percentage) | 53 (16.31%) | 22 (13.8%) | **P= 0.52^2^** |

**^1^** unpaired, two-tailed t-test

2 Chi^2^ test

**Supplemental Table 2 - Fractional anisotropy in thalamic nuclei and their association with postoperative delirium**

| *Fractional Anisotropy* | *Left* | | | | *Right* | | | |
| --- | --- | --- | --- | --- | --- | --- | --- | --- |
|  | *OR* | *95% CI* | *adj* | *95% CI* | *OR* | *95% CI* | *adj* | *95%CI* |
| *hemisphere* | 0.92 | 0.68-1.23 | 1.06 | 0.76-1.47 | 0.86 | 0.65-1.15 | 1.01 | 0.68-1.50 |
| *Pulvinar* | 1.08 | 0.80-1.45 | 1.24 | 0.91-1.71 | 0.88 | 0.65-1.18 | 0.93 | 0.67-1.28 |
| *anterior* | 0.84 | 0.63-1.14 | 0.95 | 0.68-1.31 | 0.85 | 0.64-1.13 | 0.98 | 0.66-1.44 |
| *Medio-dorsal* | 0.85 | 0.63-1.15 | 0.90 | 0.65-1.26 | 0.81 | 0.60-1.09 | 0.88 | 0.61-1.28 |
| *Ventral-latero-dorsal* | 1.23 | 0.91-1.66 | 1.34 | 0.98-1.82 | 0.96 | 0.71-1.29 | 1.10 | 0.79-1.53 |
| *Central-Lateral/ Lateral-Posterior/ Medial-Pulvinar* | 0.93 | 0.70-1.25 | 1.01 | 0.73-1.39 | 0.92 | 0.68-1.24 | 1.04 | 0.74-1.48 |
| *ventral-anterior* | 0.95 | 0.70-1.27 | 1.06 | 0.77-1.47 | 0.999 | 0.74-1.34 | 1.23 | 0.85-1.79 |
| *Ventral-latero-ventral* | 0.99 | 0.74-1.33 | 1.09 | 0.80-1.48 | 1.00 | 0.74-1.35 | 1.11 | 0.81-1.52 |

Note: Table showing the results of multivariable logistic regressions, each of them with the fractional anisotropy of different thalamic nuclei or thalamic regions obtained by diffusion tensor imaging. Effect sizes are given as odds ratio (OR) per standard deviation increment and separately for each hemisphere (Left; Right). 95% confidence intervals (95% CI) are shown in columns. Adjusted multivariable logistic regression (adj) was run together with the adjustment variables age, sex and study site. Results with significant CIs are listed in bold numbers.

**Supplemental Table 3 - Mean kurtosis in thalamic nuclei and their association with postoperative delirium**

| *Mean Kurtosis* | *Left* | | | | *Right* | | | |
| --- | --- | --- | --- | --- | --- | --- | --- | --- |
|  | *OR* | *95% CI* | *adj* | *95% CI* | *OR* | *95% CI* | *adj* | *95%CI* |
| *hemisphere* | 0.93 | 0.70-1.24 | 0.93 | 0.68-1.26 | 0.91 | 0.68-1.21 | 0.85 | 0.61-1.17 |
| *Pulvinar* | 0.91 | 0.68-1.22 | 0.91 | 0.67-1.24 | 1.05 | 0.79-1.41 | 1.12 | 0.82-1.51 |
| *anterior* | 1.01 | 0.75-1.35 | 1.01 | 0.73-1.40 | 1.03 | 0.77-1.39 | 0.97 | 0.68-1.37 |
| *Medio-dorsal* | 0.94 | 0.70-1.25 | 0.89 | 0.64-1.24 | 0.77 | 0.58-1.04 | 0.68 | 0.48-0.96 |
| *Ventral-latero-dorsal* | 1.02 | 0.76-1.37 | 1.05 | 0.77-1.43 | 0.98 | 0.73-1.31 | 0.91 | 0.65-1.26 |
| *Central-Lateral/ Lateral-Posterior/ Medial-Pulvinar* | 0.88 | 0.65-1.19 | 0.80 | 0.58-1.12 | 0.92 | 0.68-1.23 | 0.83 | 0.59-1.16 |
| *ventral-anterior* | 1.11 | 0.80-1.53 | 1.09 | 0.77-1.54 | 0.995 | 0.74-1.34 | 0.92 | 0.67-1.25 |
| *Ventral-latero-ventral* | 0.90 | 0.73-1.32 | 1.06 | 0.77-1.45 | 1.01 | 0.75-1.36 | 1.04 | 0.76-1.42 |

Note: Table showing the results of multivariable logistic regressions, each of them with the mean kurtosis of different thalamic nuclei or thalamic regions obtained by diffusion tensor imaging. Effect sizes are given as odds ratio (OR) per standard deviation increment and separately for each hemisphere (Left; Right). 95% confidence intervals (95% CI) are shown in columns. Adjusted multivariable logistic regression (adj) was run together with the adjustment variables age, sex and study site. Results with significant CIs are listed in bold numbers.

**Supplemental Table 4 - Free water in thalamic nuclei and their association with postoperative delirium**

| *Free Water* | *Left* | | | | *Right* | | | |
| --- | --- | --- | --- | --- | --- | --- | --- | --- |
|  | *OR* | *95% CI* | *adj* | *95% CI* | *OR* | *95% CI* | *adj* | *95%CI* |
| *hemisphere* | **1.43** | **1.09-1.89** | 1.41 | 0.99-2.00 | **1.38** | **1.05-1.82** | 1.39 | 0.93-2.08 |
| *Pulvinar* | 1.28 | 0.97-1.69 | 1.18 | 0.87-1.59 | **1.45** | **1.07-1.96** | 1.37 | 0.99-1.88 |
| *anterior* | 1.33 | 0.999-1.78 | 1.23 | 0.88-1.74 | 1.18 | 0.89-1.57 | 1.01 | 0.65-1.57 |
| *Medio-dorsal* | **1.40** | **1.06-1.84** | 1.37 | 0.98-1.93 | **1.36** | **1.03-1.80** | 1.31 | 0.91-1.90 |
| *Ventral-latero-dorsal* | 1.27 | 0.98-1.66 | 1.19 | 0.87-1.62 | 1.26 | 0.96-1.66 | 1.12 | 0.80-1.57 |
| *Central-Lateral/ Lateral-Posterior/ Medial-Pulvinar* | 1.23 | 0.95-1.60 | 1.16 | 0.86-1.57 | 1.18 | 0.89-1.56 | 1.10 | 0.76-1.58 |
| *ventral-anterior* | 1.32 | 0.998-1.74 | 1.22 | 0.87-1.70 | 1.22 | 0.92-1.61 | 1.07 | 0.73-1.57 |
| *Ventral-latero-ventral* | 1.24 | 0.93-1.65 | 1.15 | 0.85-1.56 | 1.17 | 0.88-1.56 | 1.06 | 0.77-1.47 |

Note: Table showing the results of multivariable logistic regressions, each of them with the fractional anisotropy of different thalamic nuclei or thalamic regions obtained by diffusion tensor imaging. Effect sizes are given as odds ratio (OR) per standard deviation increment and separately for each hemisphere (Left; Right). 95% confidence intervals (95% CI) are shown in columns. Adjusted multivariable logistic regression (adj) was run together with the adjustment variables age, sex and study site. Results with significant CIs are listed in bold numbers.

**Supplemental Table 5 - Sensitivity Analysis**

| *Thalamic Diffusion Metric* | *OR adjusted (95% CI)* | *p-value* |
| --- | --- | --- |
| *Fractional Anisotropy* | 0.88 (0.57-1.35) | 0.55 |
| *Mean Diffusivity* | **1.84 (1.22-2.76)** | **0.003** |
| *Mean Kurtosis* | 0.70 (0.47-1.03) | 0.07 |
| *Free Water* | 1.72 (1.09-2.73) | 0.02 |

Note: Table showing the results of multivariable logistic regressions (n=223), each of them with a different diffusion tensor imaging metric of the thalamus as predictor variable and their odds ratio (OR) for postoperative delirium. Effect sizes are given as odds ratio (OR) per standard deviation increment. 95% confidence intervals (95% CI) are shown in parentheses. Adjusted multivariable logistic regression (OR adjusted) was run together with the adjustment variables age, sex, study site, type of anaesthesia (general, regional combined), type of surgery and duration of anaesthesia (in minutes). After a Bonferroni correction the significance level was set to p < 0.0125 for the original analysis and was also applied to the sensitivity analysis. Significant results are in bold numbers.
